# Nanoscale and Macroscale Scaffolds with Controlled-Release Polymeric Systems for Dental Craniomaxillofacial Tissue Engineering

**DOI:** 10.3390/ma11081478

**Published:** 2018-08-20

**Authors:** Saeed Ur Rahman, Malvika Nagrath, Sasikumar Ponnusamy, Praveen R. Arany

**Affiliations:** 1 Departments of Oral Biology and Biomedical Engineering, School of Dentistry, University at Buffalo, Buffalo, NY 14214, USA; saeedbio80@gmail.com (S.U.R.); mnagrath@ryerson.ca (M.N.); sponnusa@buffalo.edu (S.P.); 2 Interdisciplinary Research Centre in Biomedical Materials, COMSATS University Islamabad, Lahore Campus, Lahore 54000, Pakistan; 3 Department of Biomedical Engineering, Ryerson University, Toronto, ON M5B 2K3, Canada

**Keywords:** nanofibers, electrospinning, stem cells, growth factors, microspheres, nanosphere, 3D additive printing

## Abstract

Tremendous progress in stem cell biology has resulted in a major current focus on effective modalities to promote directed cellular behavior for clinical therapy. The fundamental principles of tissue engineering are aimed at providing soluble and insoluble biological cues to promote these directed biological responses. Better understanding of extracellular matrix functions is ensuring optimal adhesive substrates to promote cell mobility and a suitable physical niche to direct stem cell responses. Further, appreciation of the roles of matrix constituents as morphogen cues, termed matrikines or matricryptins, are also now being directly exploited in biomaterial design. These insoluble topological cues can be presented at both micro- and nanoscales with specific fabrication techniques. Progress in development and molecular biology has described key roles for a range of biological molecules, such as proteins, lipids, and nucleic acids, to serve as morphogens promoting directed behavior in stem cells. Controlled-release systems involving encapsulation of bioactive agents within polymeric carriers are enabling utilization of soluble cues. Using our efforts at dental craniofacial tissue engineering, this narrative review focuses on outlining specific biomaterial fabrication techniques, such as electrospinning, gas foaming, and 3D printing used in combination with polymeric nano- or microspheres. These avenues are providing unprecedented therapeutic opportunities for precision bioengineering for regenerative applications.

## 1. Introduction

The field of regenerative medicine has banked on the significant advancements in various disciplines in science from engineering, biology, and medicine. Research has focused on enhancing the health of the patients through regeneration of damaged or diseased tissues and organs. Given our understanding of stem cells, regenerative medicine is exquisitely focused on directing cellular behavior to evoke therapeutic clinical outcomes [1,2,3]. Biomaterial scaffold systems have been extensively utilized for these purposes for various tissues and organs, such as the cornea, skin, bone, muscle, neural, and dental tissues. Craniofacial tissue engineering has focused on generating biomaterial systems to promote development of specific oral and dental tissues, such as bone, dentin, cementum, periodontal ligaments, mucosa, and salivary glands [4]. It is well known that cells respond to the chemical and topographical cues from their microenvironment [2,5]. 

**Figure 1:**
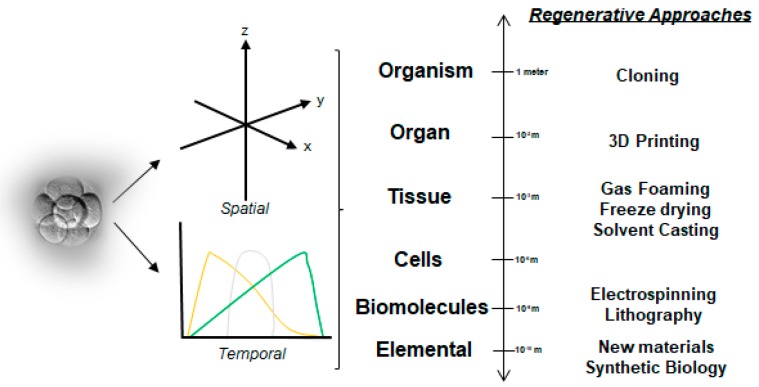
Outline of directed differentiation strategies for tissue engineering that utilize precision-engineered physical (insoluble) and biochemical (soluble) cues in a spatiotemporally regulated manner. Growing emphasis on the hierarchical (discrete scales) modular design and synthesis of biomaterial systems has further improved overall functionality and utility.

Efforts have focused on generating scaffold systems that mimic the native physical environment, as well as provide instructional biochemical cues to promote optimal functions. The complexity of biological systems are clearly evident during development as a mass of undifferentiated embryonic cells increase their numbers, migrate, elaborate extracellular matrix (ECM), and differentiate to form tissues and organs (Figure 1) [6]. The exquisite roles of soluble regulatory biomolecules in these inductive processes have enabled their applications in specific clinical contexts. The major objective of this narrative review provides a brief overview of discrete biomaterial techniques, namely electrospinning, gas-foaming, and 3D additive printing, in combination with polymeric encapsulation techniques, highlighting our research efforts wherever appropriate, to develop sophisticated, precision-engineered biomaterial scaffold systems for tissue regeneration.

## 2. Fundamental Principles of Engineering Tissues and Organs

The major premise of tissue engineering is to mimic the natural process of embryonic development, where undifferentiated cells are directed to form functional tissues and organs. Advances in our basic understanding of biology, as well as advances in biomaterials, including fabrication and nanotechnologies, are heralding a rapid progress in tissue engineering. 

### 2.1. A Simplistic View of the Time–Space Paradigm in Tissue Engineering

**Figure 2:**
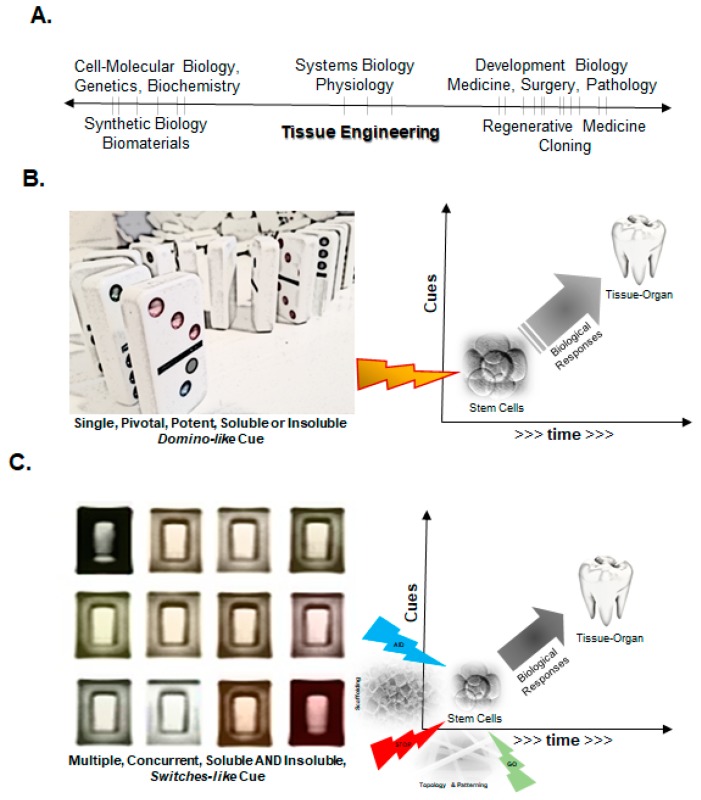
Fundamental principles of tissue engineering applied to current progress in medicine and biology. (**A**) Various fields in biology, biomaterials, and medicine that span fundamental lab research and applied clinical translation. Tissue engineering is uniquely poised to enable bridging the gap between basic and applied sciences to improve human health; (**B**) Domino model of directing stem cell differentiation and promoting tissue-organ generation by utilizing a single, pivotal potent cue that promotes directed biological responses; (**C**) The switchboard model of directed differentiation of stem cells that utilizes multiple, “switch”-like cues that promote functional generation of tissues or organs. Both models can utilize soluble (biological) and insoluble (matrix) cues.

Developmental biologists have been examining the earliest steps of the embryo to decipher a thorough understanding of the cell fate determination and tissue patterning. These top-down investigations have informed the field of stem cell biology, enabling remarkable progress in lineage reversals (induced pluripotency). A major emphasis of these explorations has focused on epigenetic—both intracellular and extracellular matrix driven—regulation. A culmination of these concepts is driving a bottom-up approach to engineer cells in the field of synthetic biology. The use of biomaterials is enabling bridging the gap between cell-tissue engineering and clinical applications in regenerative medicine (Figure 2A). The fundamental biological principles driving these engineering efforts to promote directed differentiation of cells, either exogenously transplanted or recruited endogenously from the host, have been focusing on providing instructional cues in a deterministic manner. These cues can be provided in a spatially and temporally discrete manner as a “domino” or “switchboard” model [6]. The domino model refers to the utilization of a single deterministic cue, either soluble biological molecule (e.g., a growth factor) or matrix topology (e.g., aligned nanofibers), capable of promoting a directed biological fate (Figure 2B). This process relies on a thorough understanding of the pathway and ultimate endpoint of the desired biological response, but relies explicitly on a single deterministic intervention that generates a homotypic tissue that subsequently promotes other tissue organizations, including vascular or nerve supply. An example of this approach is the use of the INFUSE device for maxillofacial reconstructions. It consists of an acellular collagen scaffold with sustained release of recombinant human bone morphogenetic protein-2 (BMP-2) to promote bone formation [7,8]. It is worth pointing out that while the collagen, in this case, serves as a carrier for the growth factor and preliminary scaffold to accommodate initial osteoinduction, it is not specifically designed to promote bone formation or growth. In contrast to the simpler domino approach, there are several scenarios where multiple cues, especially spatial conformation, are critically necessary to incite a concerted, therapeutic biological response. This is analogous to a switchboard-like manner where precisely engineered soluble and insoluble cues are provided concurrently to induce and direct cellular responses to form tissues and organs (Figure 2C). The generation of heterotypic tissues, such as multiple (support) cell types, vasculature or innervation, appears to be a key design principle for generation of the functional end organ. An example of this approach is 3D bioprinted tissues, where a combination of cells and factors are generated in physiologically relevant configurations [9,10]. Both engineering strategies are being effectively utilized currently, based on the extent and complexity of the functional tissues needed. 

### 2.2. Biomaterial Fabrication Approaches

The two major goals of tissue engineering are to provide an optimal physical and biochemical microenvironment to promote directed differentiation and maintenance of a mature, physiologically competent new tissue or organ. Tremendous progress in material processing and fabrication has led to our ability to exquisitely accomplish both these major design criteria. Biomaterial techniques can be broadly categorized as additive or subtractive, and can range from large (centi- or millimeter) to miniscule (micro- or nanometer) scales. Scaffolds can be generated using a variety of techniques, such as phase separation, template synthesis, self-assembly, solvothermal synthesis, inverse gas chromatography, solution phase growth, gas foaming, wet spinning, stereolithography, selective laser sintering, and three-dimensional printing [11,12,13,14,15,16,17]. 

To mimic the natural cellular milieu, biomaterial approaches have been used to simulate the physical (insoluble) and biochemical (soluble) biological microenvironments. Electrospinning technique has been popular since its early development in the 1930s [18]. It has recently regained much interest due to its ability to generate nanoscale features mimicking the natural ECM that impacts cell survival, shape, and reorganization [19,20,21,22]. Electrospun nanofibrous scaffolds have several desirable properties, such as high surface area, protein absorption, customized contiguity, binding sites for cellular interactions, and activation of specific intracellular signaling and gene expression, among others. However, electrospinning alone has been noted to have some limitations. First, besides a physical nanoscale ECM-simulating niche, several soluble biomolecules are usually necessary to create a favorable microenvironment to promote directed cell responses [23,24]. Microencapsulation techniques can be combined with electrospinning where active biomolecules within polymeric spheres or liposomes are utilized to enable controlled and sustained delivery systems [25,26,27,28]. Second, a major challenge is the mechanical strength and compliance of electrospun scaffolds. Third, several biological structures require hierarchical assembly of nanostructures into micron milli- or centimeter scale functional arrangements. These latter limitations are being addressed by microfabrication approaches, such as gas foaming, lithiography, microfluidics, or 3D printing [29,30,31,32]. Overall, there are a broad range of topics that encompass the current tissue engineering advances. However, this narrative review largely focuses on a few design principles and specific efforts at promoting dental and craniomaxillofacial tissue engineering. 

## 3. Material Selection for Biomaterial Systems

Biomaterial advances have enabled significant improvements in our daily lives, from automotive, fabrics, and agriculture to biology and medicine. These biomaterial technologies have essential components—the choice of biomaterial itself and its fabrication process. The choices of a biomaterial are imperative to the design and eventual function in a given biological scenario. Polymers are very popular as they can be fabricated into adaptable scaffolds, such as gels and fibers, are biocompatible and biodegradable, and can be further functionalized chemically on their surfaces to improve specific bioactivity. Biodegradable polymers are attractive candidates for scaffolding materials because they degrade and turn over with the new tissue formation. The major challenges in scaffold manufacture lie in the design and fabrication of customizable biocompatible and biodegradable constructs with properties that promote cell attachment, proliferation, and differentiation, along with sufficient mechanical properties that match the host tissue, with a predictable degradation rate and biocompatibility. Polymeric scaffolds play vital role in tissue engineering through cell proliferation, differentiation, and new tissue formation, showing great promise in the research of engineering in a variety of manners. Surface micro- or nano-topography, surface area, porosity, and pore size are widely considered as important parameters for tissue engineering scaffolds. These features are suggested to be essential for cell adhesion, proliferation, migration, differentiation, and tissue formation [33,34]. These features allow a polymer scaffold to be used into biological systems, and designed to mimic the natural ECM microenvironment.

Broadly, there are two kinds of polymers used for these applications: naturally derived and synthetic polymers. Some examples of natural polymers include collagen, alginate, fibrin, silk, and chitosan. A few major limitations of these polymers are potent immunogenicity, difficult to process at large scales, and variability due to their natural source. Therefore, these liabilities have prompted the development of synthetic polymers with favorable characteristics [35]. Synthetic polymers are attaining popularity because they have a high mechanical stability, processing capability, biocompatibility, and biodegradability [36]. The most popular synthetic polymers for three dimensional scaffolds in tissue engineering are saturated poly(α-hydroxy esters), including poly(lactic acid) (PLA), poly(glycolic acid) (PGA), poly(lactic acid-co-glycolic acid) (PLGA), and poly(ɛ-caprolactone) (PCL). PCL and PLGA are widely used in tissue engineering for treating patients suffering from damaged tissue or lost organs [37,38]. These materials have excellent biocompatible and biodegradable properties that are Food and Drug Administration (FDA) approved for clinical use. Besides polymers, several tissue engineering applications have utilized natural and synthetic biomaterials, such as ceramics, proteins, and metals [39,40,41,42,43]. Metal-based scaffolds have been used specifically for bone engineering due to their mechanical properties and ability to promote bone growth. Commonly employed materials include tantalum (Ta) and titanium (Ti) that demonstrate improved osteoblast adhesion, proliferation, and differentiation [44,45]. 

## 4. Polymeric Microencapsulation for Nano- and Microspheres

Microencapsulation methods in pharmacology have enabled controlled and precise delivery of a range of molecules that increase sustained and therapeutic dosage, concurrently reducing side effects. Formulations often utilize polymeric encapsulation as nano- or microspheres. Popular synthetic polymers for these applications include polyesters and acrylic derivatives. Polyesters are synthetic polymers that are degraded in the body by hydrolysis. Some common examples are poly-ε-caprolactone (PCL) and poly(lactic-co-glycolic) acid (PLGA) that are used to deliver various drugs [46,47,48]. By using specific polymer compositions that determine degradation rates, long-term controlled drug release can be achieved over weeks to months [49]. 

**Figure 3:**
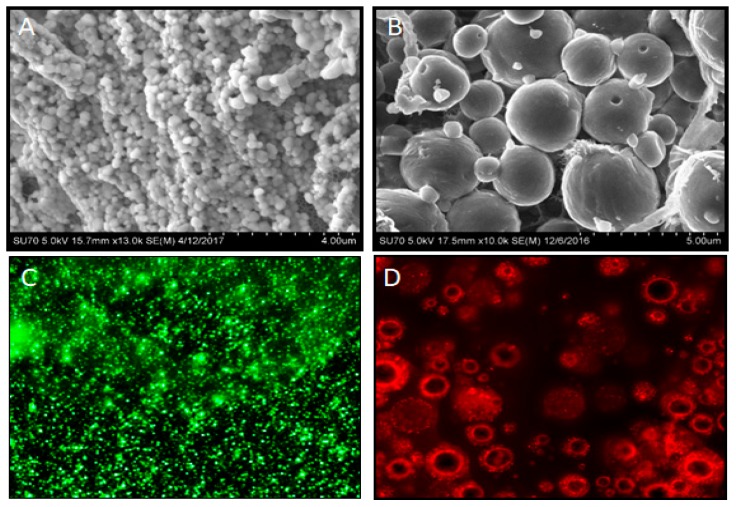
Controlled release systems for bioactive agents can be effectively achieved with polymeric encapsulation into nano- or microspheres. The images show scanning electron microscopy images of poly(lactic-co-glycolic) acid (PLGA) microspheres at low (**A**) and high (**B**) magnifications. As proof of principle, fluorescein (**C**) green, or hematoporphyrin (**D**) red, payloads in PLGA microspheres were imaged using a fluorescent microscope.

A wide variety of therapeutic molecules, such as growth factors and soluble small molecules, have been encapsulated in PLGA formulations [50,51]. The interaction between the polymer and the encapsulated drug, and the cellular environment, is influenced by the surface morphology of microspheres. Many different methods, such as emulsion freeze-drying, electrospraying, nanoprecipitation, and hydrolysis, can be utilized for the drug encapsulation. Among them, single and double emulsion are the most popular methods to prepare these polymeric spheres with different morphologies including solid, porous, or hollow structures [52,53,54]. PCL has been studied in many novel drug delivery systems and tissue engineering applications [55,56]. It is well known that soluble factors, including growth factors, small molecules, and cytokines, can exert robust effects on cell fate. Recently, we demonstrated the use of PLGA microspheres generated by solvent evaporation method using a double emulsion technique to deliver multiple growth factors to direct stem cell differentiation (Figure 3) [57]. A key advance in these systems is the use of an antagonistic cue that aided in the development of sharp morphogen fields allowing lineage-restricted stem cell differentiation. 

## 5. Electrospinning to Generate Nanofiber Scaffolds

During the past decade, several techniques have been applied for synthesizing nanofibrous scaffolds. Among the most common are temperature-induced phase separation, particulate leaching, phase inversion in the presence of a liquid non-solvent, emulsion freeze-drying, electrospinning, and rapid prototyping [35,58,59,60,61,62]. On the other hand, foaming of polymers using supercritical fluids is a versatile technique in obtaining porous structure [63]. Electrospinning is a popular technique for polymer processing to generate non-woven matrices with nanoscale features. The basic principle of the electrospinning method is to generate nanofiber sheets from a polymer solution extruded into a strong electric field. The high voltage reduces surface tension within the polymer fluids, enabling generation of nanoscaled fibers. The thickness of individual fibers, their orientation, and the overall thickness of the matrix sheet, can be controlled through the type of solvents, polymer concentrations, surfactants, type of collector, distance, and time for electrospinning. 

### 5.1. Nanofiber Scaffold

Most biomaterials, both synthetic and natural polymers, used in tissue engineering, have been fabricated into the nanofibers via electrospinning technique [42,64]. Due to their excellent biomechanical, especially thermal stability, and biocompatible properties, most studies have focused on PCL nanofiber. A major feature of these PCL scaffolds is substantial elongation and small-sized fibers mimicking the natural ECM [65]. Nanostructures of the ECM play critical roles in directing cellular behavior and functions [66]. The natural ECM, consisting of predominantly collagen fibrils, along with elastin and ground substance, creates a favorable physical microenvironment for cell adhesion, proliferation, and differentiation [38,67]. The polymeric non-woven, electrospun nanofiber scaffolds mimic these effectively with their high porosity and high surface area [64]. The high surface area to volume ratio enables optimal cell adhesion, while the high porosity enables competent nutrient transport, making this technique ideal for several tissue engineering applications. These nanofiber scaffolds have been shown to promote specific cellular functions, such as cell adhesion, proliferation, differentiation, and modulation of stem cell fate for tissue regeneration [36,61,68]. 

### 5.2. Roles of Extracellular Nanostructures in Cell Differentiation

Normal cells, with the exception of hematopoietic lineage, require a continuous flow of signals from their adhesion to ECM to ensure survival. These exogenous signals are relayed via receptor-mediated signaling that epigenetically regulates proliferation, differentiation, and various functions. Another key function of the ECM is to immobilize and present various biomolecules, such as growth or differentiation factors, regulatory nucleic acids, glycoproteins, and lipids that can, themselves, modulate cell phenotype [69]. Besides these critical roles, the ECM also enables cell-to-cell interactions by providing a foundational matrix. The electrospun nanofibers have been shown to stimulate these functions effectively. Nanofiber structures promoting directed differentiation of stem cells have been noted in a broad range of cell fates, namely osteoblasts [70], odontoblasts [61], endothelial cells [71], neural cells [72], fibroblasts [73], macrophages [74], and osteoblast-like cells [75]. A report demonstrated osteogenic differentiation of human mesenchymal stem cells (hMSCs) in these nanofiber scaffolds in the absence of osteogenic induction media that can be further enhanced with these supplements [76,77]. Other reports have examined its role in mineralized tissue regeneration, where the nanofiber architecture was noted to specifically promote an osteoblast or odontoblast MSC fate, resulting in cell differentiation and biomineralization in vitro and in vivo [43,61]. Interestingly, these scaffolds not only promoted differentiation of MSCs, but also promoted functional elaboration of matrix and mineralization of pre-existing osteoblasts (bone) and odontoblasts (dentin). Most notably, some of these stem cell responses to the nanofiber scaffolds appear to occur independent of exogenous growth factors, suggesting direct effects of nanotopology on cell responses [61]. 

### 5.3. Microsphere- or Nanosphere-Incorporated Nanofibrous Scaffolds

**Figure 4:**
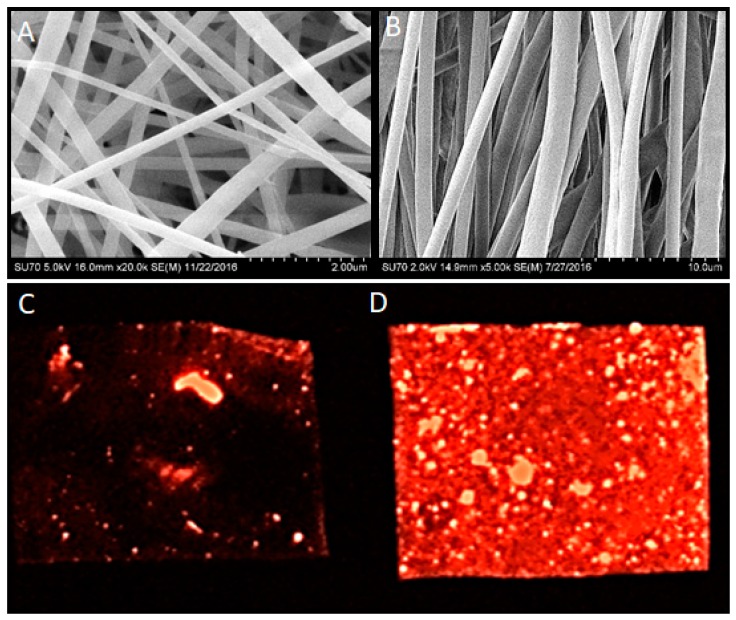
Electrospun nanofiber scaffolds were generated with PCL and imaged with scanning electron microscopy. Based on specific electrospinning conditions, nanofibers can be either randomly organized (**A**) or linearly aligned (**B**). Incorporation of a red fluorescent dye during electrospinning leads to concentration-dependent distribution, as shown at low (**C**) and high (**D**) magnifications imaged with a fluorescent microscope.

Besides the nanotopology of these nanofiber scaffolds, the ability to immobilize and present biological cues appears to be critical to fully mimic the natural ECM. There have been several attempts at combining biomaterial scaffolds with controlled-release systems for specific applications [74]. Some approaches have simply utilized the addition of payloads to polymers prior to electrospinning, while others have used polymeric microspheres for encapsulation that are admixed with polymers prior to electrospinning [78,79,80]. The payloads in these approaches have varied from growth factors to small molecules (drugs) that have specific clinical applications (Figure 4). Combinations of a specific payloads and architecture (aligned) nanofiber scaffolds are enabling use of electrospun scaffolds for neural bioengineering, dental and craniofacial applications, and wound dressings, among others [81]. 

## 6. Gas Foaming and Water Leaching

**Figure 5:**
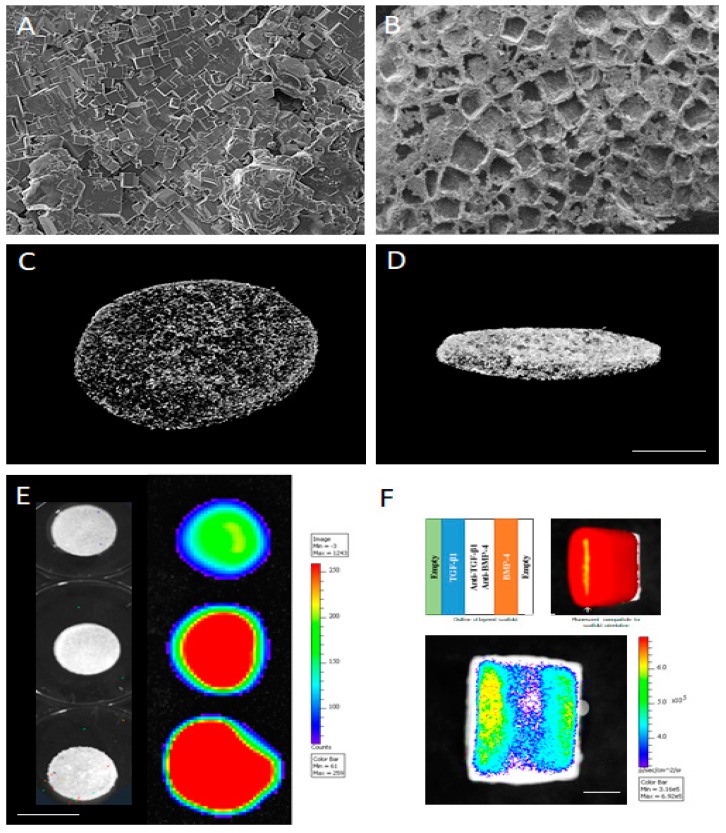
PLGA can be fabricated into scaffolds using gas foaming technique with a water-soluble porogen, salt particles in this case. Scanning electron microscope shows polymer–salt mix after foaming (**A**) and water leaching (**B**), demonstrating the uniform pore network. The interconnectivity of the scaffold pore network can be assessed with microcomputed tomography, as seen from the top (**C**) and side (**D**) Scale bar = 1 cm. These scaffolds can be fabricated with the PLGA microspheres that release specific payloads, as shown here with a morphogen alone, TGF-β1, in a dose-dependent manner (**E**). These strategies can be combined with multiple morphogens, TGF-β1 and BMP-4, in this case, along with their pathway-specific inhibitor such as a TGF-β inhibitor, SB43152, and BMP inhibitor Dorsomorphin. Scale bar = 3 cm (**F**) to create spatially-restricted, morphogen fields within scaffolds. Scale bar = 1.5 cm.

Another popular scaffold fabrication technique is the use of high pressures to melt and foam polymers around precisely sized porogens. This technique was developed to overcome the limitations of solvent casting that use organic solvents. Gas foaming utilizes high pressure carbon dioxide (CO_2_) for protracted periods (16 h to couple of days) around a polymer and porogen (usually sugar or salt particles). The CO_2_ gas is incorporated into the polymeric material, and when the pressure is released in a controlled manner, the resultant foaming process ensures polymer flows around the porogen and forms a porous scaffold structure. The porogen is then leached by simply putting the scaffold construct in water, forming a sponge-like structure (Figure 5). The limitation of this technique is that the polymer and payloads within are subjected to excessive heat and pressure during compression molding, and have limited pore interconnected structures. Based on the size of the porogen, either nano- or microscale pores can be generated. Moreover, the use of PLGA microspheres enables delivery of both morphogens and their inhibitors that allows generation of exquisite spatiotemporally-engineered morphogen fields. Motivated by embryonic development, these morphogen fields operate in simple (domino) or more complex (switchboard) models that provide an optimal microenvironment to direct cell fate and responses [6]. Fang et al. reported microspheres carrying transforming growth factor-β1 (TGF-β1) loaded into chitosan bilayer membrane-induced dentin regeneration in beagle dogs [75]. We had previously reported using TGF-β1, TGF-β3, and BMP4 within PLGA microspheres to generate dentin, cartilage, and bone [82]. 

The use of specific morphogens was capable of promoting lineage-restricted differentiation of dental and mesenchymal stem cells to dentin, bone, or cartilage by using specific growth factors, additional supplements, and altering pore size to restrict diffusion and simulate hypoxia (avascular cartilage) [82]. Using a latent growth factor complex, we were able to demonstrate temporal control within restricted morphogen spatial fields as well [57]. These strategies are able to generate discrete morphogen fields within a biomaterial scaffold system that enables complex, heterogeneous tissue differentiation from transplanted or infiltrating host cells. This is particularly attractive in practical clinical applications where multiple tissues will need to be generated to promote optimal tissue or organ functions. The use of specific morphogens was capable of promoting lineage-restricted differentiation of dental and mesenchymal stem cells to dentin, bone, or cartilage by using specific growth factors, additional supplements, and altering pore size to restrict diffusion and simulate hypoxia (avascular cartilage) [82]. Using a latent growth factor complex, we were able to demonstrate temporal control within restricted morphogen spatial fields as well [57]. These strategies are able to generate discrete morphogen fields within a biomaterial scaffold system that enables complex, heterogeneous tissue differentiation from transplanted or infiltrating host cells. This is particularly attractive in practical clinical applications where multiple tissues will need to be generated to promote optimal tissue or organ functions. 

## 7. 3D Printing

A major limitation of the biomaterial approaches described thus far is a lack of mechanical strength and inability to generate larger, clinically viable tissue or organ replacements. There has been tremendous progress with 3D printing technologies that provide significant advantages in fabricating patient-specific constructs when combined with digital imaging (optical or radiographic). 3D printing approaches can be broadly categorized as subtractive or additive techniques. Techniques for subtractive printing have been more advanced compared to more recent innovations in additive 3D printing. Nonetheless, both approaches have demonstrated significant utility in many manufacturing fields, and specifically, have shown great promise for regenerative medicine [9,83]. Additive printing has shown several significant advantages, such as a flexible manufacturing process that supports fast and accurate fabrication of complex 3D structures over a broad range of sizes ranging from submicrometer to several meters [84]. Other benefits supported by this technology include reliability, cost-effectiveness, biocompatibility, and ease of use. There are several approaches for 3D additive printing, such as fused deposition modeling, selective laser sintering, stereolithography, and 3D plotting, direct-write, or bioprinting [16]. Fused-deposition additive 3D printing technique is most popular and can be broadly categorized as laser-assisted, inkjet or extrusion-based [85,86,87]. Extrusion-based 3D printing systems are most popular as they can be used with a wide range of biomaterials. The equipment is relatively inexpensive, consisting of computer-controlled heated extruders and XYZ mechanical stages, and allows rapid custom fabrication. Generally, 3D-printed biomaterials range from cell-supportive hydrogels, to ceramic implants of metal and from quantum dots or nanoparticles for drug delivery and imaging systems, to complex functioning medical devices [15,16]. As outlined previously, 3D-printed scaffolds are essentially able to mimic ECM and simulate basic features, including porosity, pore dimensions, interconnectivity, internal geometry, mechanical properties, biocompatibility, and biodegradation kinetics. These materials should have rheological features to allow extrusion and solidification upon deposition into mechanically resilient 3D-printed structures. 

Transplantable tissues and organs are a critical healthcare challenge worldwide. Additive 3D printing offers significant promise in enabling scaffolds to generate internal and external tissues or organs, and address the shortage of transplantable organs [17]. This technology has been used successfully to generate hard tissues like teeth, bone, and cartilage, and soft tissue like skin, muscle, and complex organs like nose, ears, heart, and liver [85,88]. Jung et al. proposed multiple-head 3D printing systems for fabricating heterogeneous cell-laden hydrogel scaffolds for the kidney, outer ear, and tooth tissue [17]. Cell-printing or bioprinting method employs living cells in the 3D construct fabrication process, together with the essential advantages of printing-based rapid prototyping. Depending on the applications, cellular bioprinting can be classified into three types: droplet-based, extrusion-based, and stereolithography [9,89]. Kang et al. developed integrated tissue–organ printer (ITOP) technology that can print human-scale tissue models, such as ear-shaped cartilage, mandible bone, and structured skeletal muscle. The ITOP system has the ability to print cell-laden hydrogels with a polymer fabricating tissue constructs with the high structural integrity necessary for clinical implantation. Their study demonstrated the possibility of printing desired living tissue constructs that mature into vascularized functional tissues in vivo. However, the potential host immune responses to transplanted scaffolds indicating long-term studies are still necessary for 3D bioprinted transplants [90]. 

### 7.1. Designing Bioactive Systems with 3D Printing

**Figure 6:**
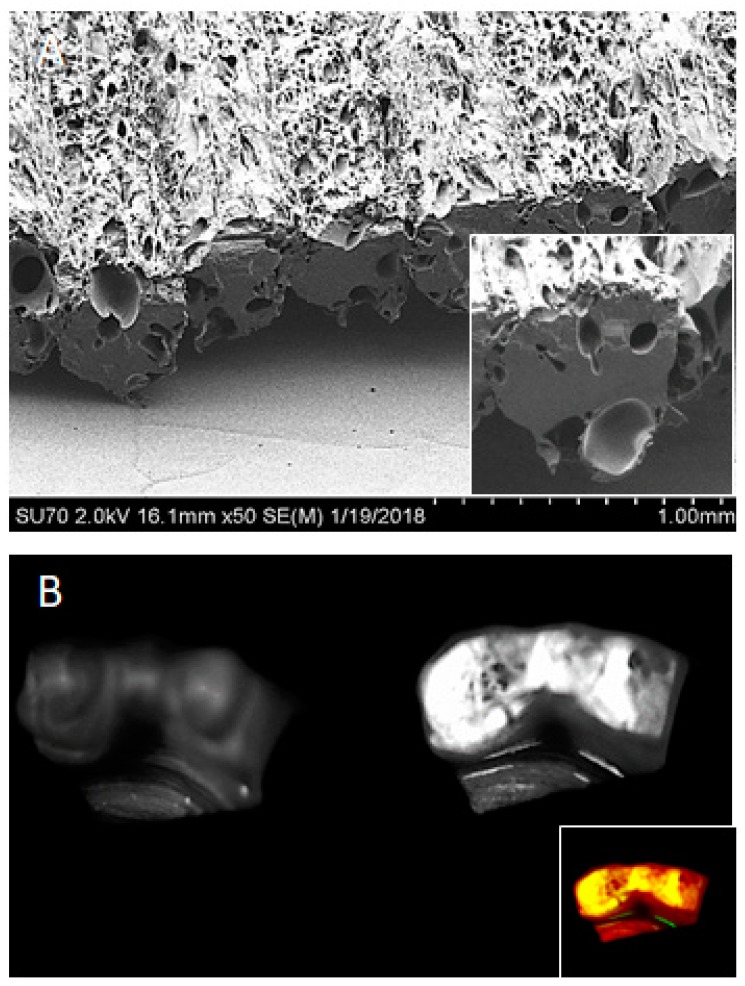
3D-printed Poly(methyl methacrylate) (PMMA) prosthesis by fused-filament fabrication with PLGA microspheres incorporated into tissue interface layer. Scanning electron microscopy demonstrates the porous PMMA surface (**A**) that contains the PLGA microspheres (high power, inset). These microspheres were synthesized with hematoporphyrin (pseudocolored inset) and imaged with a fluorescence gel doc reader (**B**).

As discussed with prior biomaterial approaches, 3D-printed biomaterials can offer the physical niche (structural and mechanical properties) mimicking natural ECM, providing a favorable microenvironment for cell adhesion, survival, migration, proliferation, and differentiation [91]. Soluble biochemical cues can also be included with 3D-printing technique, where selective deposition of peptides, proteins, and regulatory nucleic acids can be achieved. Simple admixture of agents into the biomaterials for printing are prone to potential damage or deterioration during the thermoplastic 3D printing process. Additionally, incorporation of the bioadditive molecules often interferes biochemically or sterically with the homogeneity of the scaffold material potentially compromising its mechanical properties. Hence, polymeric microencapsulation techniques in the form of nano- or microspheres provide a useful approach to incorporate various bioactive agents. Shim et al. demonstrated the utility of rhBMP-2-loaded polycaprolactone/poly(lactic-co-glycolic acid)/β-tricalcium phosphate (PCL/PLGA/β-TCP) membranes fabricated by 3D printing for guided bone regeneration [92]. Fahimipour F et al. fabricated a vascular endothelial growth factor (VEGF)-loaded gelatin/alginate/β-TCP composite scaffold by 3D printing to promote craniofacial tissue engineering [93]. Our group recently demonstrated feasibility of this approach with small molecules in PLGA microspheres imparting anti-fungal characteristics to polymethylmethacrylate dental prosthesis (Figure 6) [94]. These strategies are enabling simulation of both soluble and insoluble natural functions of the ECM. 

### 7.2. Sense-and-Respond “Smart” Biomaterials for Theranostics

**Figure 7:**
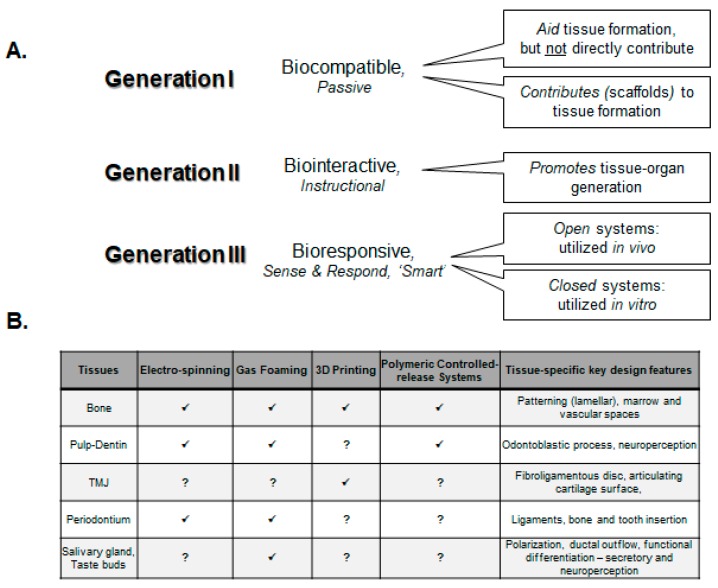
Development of biomaterials for tissue engineering applications. (**A**) Outline of biomaterials properties as they have evolved to provide increased functions in various lab and clinical regenerative context; (**B**) Dental and craniomaxillofacial tissue engineering efforts highlighting various material fabrication approaches and unique design criteria for individual applications.

The polymeric microspheres described previously can not only deliver biological payloads, but also serve diagnostic sensing functions [95,96,97]. The use of specific sense-and-respond “smart” systems are playing a key role in the *theranostics*, a term referring to diagnostics and therapy (Figure 7A). Chen J et al. used sorafenib-eluting PLGA microspheres for delivery by intrahepatic transcatheter infusion [98]. These microspheres also included iron oxide nanoparticles, enabling magnetic resonance imaging (MRI) of intrahepatic biodistributions. Thus, targeted distribution and delivery of a bioactive agent was feasible. You J et al. used the photothermal effects mediated by a near-infrared (NIR) laser and hollow gold nanospheres (HAuNSs) to release an anticancer agent, paclitaxel (PTX), from PLGA microspheres [99]. NIR treatments not only resulted in photothermal damage to tumor cells, it also released PTX from the microspheres, resulting in synergistic, significant destruction of tumor cells. 

Similar strategies in biomaterial scaffold systems can be envisioned that enable an exquisite feed forward, stimuli-responsive bioavailability of biological payloads from these microsphere systems in 3D-printed scaffolds. Dentistry has also made several striking advances with smart materials [100]. These materials are designed to sense various external stimuli, such as mechanical stress, temperature, pH, or moisture. Some examples include restorative materials (composites, glass ionomers, and ceramics), implants, orthodontic appliances, surgical ligatures, and dental instruments (burs, files), among others. A further iteration of these sense-and-response strategies is development of closed loop microphysiological in vitro systems, also known as *organs-on-a-chip* [101,102,103]. These constructs consist of interconnected sets of two or more 3D cellular constructs that perform specific tissue or organ functions. While current organs-on-chip methods have relied on multistep lithographic systems and lack integrated sensors, 3D printing with theranostics microsphere systems offers significant advantages, such as the ability to create and retain heterogeneous 3D tissue constructs with small fluid volumes, and the ability to determine accurate, functional scaling of organ sizes, and topography with minimal cellular units to generate desired organ functions. 

There have been few efforts in developing microsystems for oral-dental applications, best highlighted by the development of a bioelectronic tongue based on a microelectrode array that utilizes patch clamp recordings of individual taste receptor cells to examine sensitivity to various taste stimuli [104]. Other investigators have developed microsystems that simulate pharmacodynamics of orally-ingested agents that are very useful in drug development and testing [105,106,107]. 3D printing approaches using multifunctional biomaterials can provide a valuable avenue for further development of organs-on-chips that currently serve as surrogate, in vitro model systems but may also serve, in the near future, as transplantable, artificial organs in vivo. Lind et al. demonstrated a new class of cardiac microphysiological system using multimaterial 3D bioprinting [108]. Six functional inks were designed based on high-conductance, biocompatible soft materials, and piezoresistance that guide the self-assembly of physiomimetic laminar cardiac tissues. These devices were used to study drug responses, and contractile development of human stem cell-derived laminar cardiac tissues over four weeks. 

## 8. Applications for Dental and Craniofacial Tissue Engineering 

One of the key survival mechanisms of the human body is the ability to heal itself. Complete and ideal healing will result in regeneration. Injury and disease generate damage and deterioration of tissue and organs that require healing-regeneration or replacements. The oral environment is specifically challenging as it is assaulted by unique mechanical, microbiological, nutritional and immunological stimuli. There has been successful studies to develop a complete tooth in animals using transplanted cells and scaffolds, but there remain significant barriers to their practical clinical translation [109,110]. By contrast, approaches to develop specific oral-dental tissues have made progress, and are more practically implementable in the clinic today. The final remaining section of this review will highlight bioengineering approaches to generate craniofacial tissues, namely bone, cartilage, pulp–dentin complex, periodontal ligaments, salivary glands, and taste buds (Figure 7B). While there has been tremendous progress in muscle and neural bioengineering, there has been little emphasis on their craniomaxillofacial applications, and hence, the reader is referred to other comprehensive reviews on this subject [111,112,113,114,115,116,117,118]. 

*Craniomaxillofacial Bone*: Globally, bone is considered the second most transplanted tissue, after skin, as a result of trauma, aging, osteoporosis, and the prevalence of bone tumors. The general concept of endogenous bone tissue regeneration with surgically placed autografts has encouraged a number of innovative biomaterial strategies attempting to mimic the natural replacement. The well-established bone-inducing potency of growth factors, such as BMPs (2 and 7) are currently available clinically to promote endogenous repair strategies. Current approaches are limited to controlled delivery capable of inducing mineralized tissue formation, but lack spatiotemporal precision necessary to pattern functional bone formation. Several materials have been used as scaffolds, including bioceramics, biocompatible metals, and biopolymers. General biomaterial design features include high porosity with 3D interconnectivity, biocompatible and biodegradable material with minimal immunogenicity, and ability to provide sustained-release of bioactive factors. 

Electrospun nanofiber scaffolds have been investigated for craniofacial bone tissue engineering capable of promoting stem cells to an osteogenic fate and mineralization in 2D and 3D cultures [119]. Several modifications to these scaffolds include chitin whiskers, hydroxyapatite, poly-3-hydroxybutyrate-co-3-hydroxyvalerate, etc. to simulate a more osteointegrative milieu [120,121,122]. Frohbergh ME et al. used chitosan–hydroxyapatite electropsun nanofibrous scaffolds to promote both an osteogenic and periosteum-like microenvironment to enable better (non-weight bearing) scaffold engraftment for maxillofacial defects [123]. Dang et al. have shown the controlled release of inductive factors (TGF-β1 and BMP-2) from microparticles for up to 5 weeks regulates osteogenesis in high-density hMSC to promote enhanced endochondral bone formation [124]. As alluded to previously, our own work has focused on utilizing a osteogenic factor (BMP-4) and its antagonist (Dorsomorphin, BMP inhibitor) to generate spatially restricted morphogen fields [82]. 3D printing technology of biomaterials has revealed that increased strength and complex morphologies can be achieved effectively [125]. Besides the significant utility of patient-specific splints, surgical guides, and pre-operative training models, 3D printing is utilizing a wide range of biomaterials, such as PCL, PMMA, ABS, and bioceramic and bioactive glass for bone-promoting scaffolds [126,127,128,129,130]. Shim JH et al. demonstrated the utility of using hBMP-2-encapsulated PLGA microspheres in PCL/β-TCP 3D-printed membranes for bone formation in calvarial defects in rabbits [92]. Hence, a combination of the biomaterial techniques described above can effectively promote clinical bone regeneration in maxillofacial cranial defects due to disease or trauma. 

### 8.1. Pulp–Dentin Tissue Engineering

Dentin is the mineralized tissue that forms the core structure in teeth. It has a tubular structure with many compositional similarities with bone. The cells that form dentin are termed odontoblasts, which secrete a premineralized collagen-rich, organic matrix termed predentin, which is eventually mineralized by apatite crystal deposition [131]. Odontoblasts are ovoid to columnar cells with a large apical extension, termed the odontoblastic process, that have neurosensory functions. The pulp tissue consists of several cell types such as fibroblasts, neurons and endothelial cells. However, a specialized group of cells, developmentally attributed to be of neural crest origin, are termed dental pulp stem cells (DPSC), capable of generating odontoblasts. These cells have similar gene expression to mesenchymal stem cells, and originate from a perivascular niche [132]. Another group of cells at the tooth periapical region, especially from exfoliated deciduous teeth, capable of odontoblast differentiation, are termed the SHED cells [133]. As the cells of the pulp function in concert with dentin, they are functionally termed the pulp–dentin complex. Several signaling pathways and transcription factors have been noted to regulate differentiation of odontoblast during tooth development. Modulation of TGF-βs, Wnt, BMPs, Shh, and FGF has been noted to disrupt tooth development and cause dental defects [134,135,136]. These demonstrations of their functional roles in dentin induction also indicates their specific utility in efforts to engineering dentin [137]. 

Extensive tooth decay can lead to irreversible inflammation and necrosis of pulp tissue, necessitating its complete removal, disinfection, and replacement with an inert material popularly called root canal therapy. Root canal therapy leaves a devitalized tooth behind, which becomes prone to tooth fracture, reinfections, and subsequent tooth loss. To address these issues, researchers have explored various pulp–dentin tissue engineering approaches. Mooney DJ et al. seeded fibroblasts isolated from human adult pulp cells onto the scaffold made up from polyglycolic acid fibers. They reported attachment and proliferation of the pulp-derived fibroblast cells with a new pulp-like tissue formation by the end of 60 days [138]. Wang et al. seeded human DPSC onto nanofiber PLLA scaffolds cultured in media containing BMP7 and supplements, where they observed robust odontoblast differentiation and dentin formation using molecular markers and mineralization assay [139]. In a follow up study, they examined the specific role of nanofibers versus solid walled scaffolds, where the former scaffolds demonstrated superior odontogenic differentiation [140]. Corderio et al. seeded the porous scaffolds in place of pulp tissue in tooth slices with SHED alone or SHED and endothelial cells [141]. These slices were implanted in the 5- to 7-week-old male immunodeficient mice for 14–28 days. They reported that newly generated tissue closely resembled the pulp tissue, and the expression of DSP (a marker for odontoblastic differentiation) was higher in the samples seeded with SHED and endothelial cells than SHED alone, but the neovascularization was not very different among both the groups. Our own efforts using a PLGA microporous scaffold and ability of near-infrared laser-activated latent TGF-β1 noted the directed differentiation of bone marrow MSCs to an odontoblastic lineage and dentin induction [57,142]. In a more recent study, Vinning KH et al. utilized a biomaterial array to identify optimal adhesive biomaterials for DPSCs that are extremely attractive as future clinical restorative materials [143]. 

The enclosed pulp–dentin complex is critically dependent on its vascular network for its viability and function. Therefore, tissue engineering approaches have focused on promoting vasculogenic differentiation, as well as providing endothelial-derived growth factors have been explored. Both SHED and DPSCs have been noted to be capable of differentiating into endothelium, and the Wnt/β-catenin pathway has been noted to play a key role [144]. In contrast to promoting endogenous, resident endothelial cell differentiation, an elegant approach focused on promoting chemotactic homing of bone marrow MSCs has been explored [145]. Kim YJ et al. noted cellularization and revascularization of subcutaneously implanted extracted root canal treated human teeth in 5- to 7-week-old male mice [146]. Collagen scaffolds with tailored biomechanical properties containing VEGF, bFGF, NGF, PDGF, and BMP-7 were noted to promote dental pulp-like tissue generation [147]. 

Combining several of these ideal attributes, nanofiber scaffolds and controlled delivery systems have been proposed for pulp–dentin engineering [148]. A recent study by Li et al. used a hierarchical nanofiber PLLA scaffold with gelatin nanospheres containing VEGF that were placed within root canals of extracted teeth and implanted in nude mice [149]. The investigators observed pulp-like tissue formation in tooth root canals with a preponderance of blood vessels, noting successful regeneration. More recently, these investigators demonstrated a 3D micropatterning process using laser-guided machining that promoted odontoblastic extensions for dentin regeneration [150]. These strategies hold great promise for future clinical approaches to pulp–dentin regeneration. 

### 8.2. Temporomandibular Joint

The temporomandibular joint (TMJ) plays a key function in enabling oral-dental function has a typical bone-cartilage interface and a synovial disc forming a complex bilateral synovial articulation. Attempts at restoring a diseased or damaged TMJ have relied on a range of biomaterial scaffold systems to specifically induce bone, cartilage, or synovial disc [151,152]. As with other tissues, cartilage induction from both chondrocytes and MSCs have been promoted with molecules such as FGF, TGF, PDGF, and IGF-1 [153,154,155]. A key limitation for cartilage regeneration has been a source of primary chondrocytes. A major challenge for chondrocytes transplanted to sites of injury or damage are associated with donor site morbidity and cell retention. Thus, the use of 3D scaffolds, alone or with translated cells, is clinically, very attractive [156]. 

A unique design principle for cartilage bioengineering is its avascular nature. In a recent paper, we demonstrated the use of nonporous scaffolds driving a hypoxic environment, demonstrated by HIF1α upregulation that promoted MSCs to a chondrogenic fate [82]. Several studies have utilized electrospinning to generate nanofiber scaffolds of PLLA, PLGA, PDLA, PVA, and PCL with MSCs to promote cartilage regeneration [157,158,159,160]. Most studies exogenously supplement growth factors to the media or site of implantation. Other approaches, as noted by Zhu et al., incorporate biological cues within microspheres during PCL nanofiber scaffold fabrication [161]. These scaffolds were treated with cold atmospheric plasma to make them more conducive to human MSC attachment and growth. 3D printing allows for even more custom fabrication of these biomaterial systems with complex topologies. Legemate K et al. describe generation of TMJ fibrocartilage using CTGF and TGF-β3 within PLGA microspheres, during PCL electrospinning, to promote MSC differentiation [162]. These strategies indicate engineering components or the complete TMJ may be clinically feasible in the near future. 

### 8.3. Periodontium Bioengineering

The periodontium consists of the tooth supportive tissues that enable mechanical, nutritional, and immunological functions. These tissues include the alveolar bone that forms the tooth socket, periodontal ligaments (PDL) that anchor the teeth to the bone and cementum, a mineralized tissue that covers the tooth root and anchors PDL on the tooth. Progress in our understanding of the embryonic origin and development of the periodontium has spurred progressive or advancing engineering attempts following disease or trauma [163,164,165,166,167,168]. This field has been largely inspired by the concepts and progress in muscle bioengineering with specific advances in aligning cells and cell sheet technologies to generate dense, collagenous constructs [169,170,171,172]. As there are no transplantation—either allografts or autografts—options for PDL, untreated injuries or periodontal disease leads to progressive degenerative changes and eventual loss of teeth. Hence, there is a clear need for scaffold systems that provide a stable physical niche, are mechanically robust, protect, and support regeneration of these structures [173,174,175]. Pinese et al. examined ligament tissue regeneration in hybrid scaffolds composed of PLA and collagen/chondroitin sulfate [176]. These scaffolds provided enhanced ligamentocyte cell adhesion and proliferation in vitro, as well as collagen fibril formation. A key feature of these scaffolds is to promote aligned cell seeding and matrix deposition to promote the ligament or tendon, a key characteristic of electrospun nanofiber scaffolds [177]. Another major feature of ligament tissue engineering is the use of cyclic bioreactors to promote aligned tissue generation. These electrospun scaffolds require mechanical characteristics amenable to these physical, cyclic stress protocols [178]. Several growth factors, such as TGF-β2 and GDF-5, have also been included in these approaches. Attempts at direct 3D printing of fibrillar collagen scaffolds have also been successfully demonstrated [179,180]. Among various payloads utilized in this particular ligament bioengineering, bioactive molecules promoting bone or cementum adhesion, such as proteins or peptides from enamel, bone, and cementum, have been attempted [166,181,182,183,184]. Moreover, as a major application of these approaches will be in the context of periodontal disease with a potent inflammatory microenvironment, attempts at neutralizing these with anti-inflammatory agents have also been used [185,186]. Interestingly enough, there have been attempts at modifying dental implant interface to promote a ligamentous fibrous interface, rather than conventional osteointegration [187,188,189]. These approaches to periodontium tissue engineering can provide valuable new clinical strategies to prevent tooth loss. 

### 8.4. Salivary Glands and Taste Bud Engineering

Saliva and taste are intimately connected, and damage or disease of salivary gland adversely affects oral health. This can lead to difficulties in eating, speaking, and tooth decay, among others. The salivary glands are often damaged by radiation treatments during cancer therapy, as well as malignancies, autoimmune disease, and medications. The salivary hypofunction eventually leads to a reduction in saliva production termed xerostomia. Presently, management of xerostomia mainly relies on artificial saliva substitutes. 

Efforts are ongoing to bioengineer salivary glands that are being motivated from parallel attempts at generating other glandular organs, such as the pancreas, breast, lacrimal, and liver. Moreover, there is evidence from studies in lower animals of conserved developmental pathways involving Wnt, BMPs, and Hedgehog signaling program driving embryonic undifferentiated epithelium to teeth and taste buds cells [190]. Bioengineering efforts to generate salivary glands, termed sialospheres, have utilized a broad range of synthetic polymers, including PLGA, PEG, chitosan, and hyaluronic acid, among others, as well as decellularized ECM [191,192,193,194,195]. There has been a concerted effort to isolate and culture, both individually and as co-cultures, various cell types in these sialospheres [196]. These include pluripotent iPS stem cells to more restricted dental follicle cells and several salivary gland cells, such as acinar, myoepithelial, and ductal cells [197,198,199]. Specific growth factors, such as IGF and EGF, and cell adhesion mediators, such as laminins, have been utilized in these systems to promote directed differentiation [200,201,202]. 

A particular requirement of these salivary gland engineering efforts has been the emphasis on functionally-critical cell polarization, as well as branching morphogenesis that have been addressed by micropatterning biomaterial techniques [203,204,205]. Joraku et al. seeded normal human salivary gland cells on to the non-woven fibrous (15 µm diameter) sheets of polyglycolic acid with 95% porosity [206]. These constructs were implanted subcutaneously in athymic mice. Retrieved scaffolds showed the generation of glandular epithelial cells which were able to produce amylase and had water channel proteins. They showed that when seeded in a 3D collagen gel scaffold in vitro, these engineered constructs were able to form differentiated functionalized salivary units containing acini and ducts. The generated structures had tight junction, water channel protein expression, and amylase production. 

There have been attempts to develop tissue engineered models of taste buds from explants and isolated individual taste cells [207,208,209,210]. These studies have specifically examined the trophic role of the nerve supply in taste bud survival and function [211,212]. Several growth factors, such as FGF and EGF, and ionic concentrations in culture, specifically extracellular calcium levels, have been noted to play key roles [213,214]. These simple culture systems have provided valuable information on the basic pathophysiology of taste bud functions. The use of the sophisticated tissue engineering approaches with exquisite precision in topology (polarization and branching), morphogen fields (directed acinar and myoepithelial differentiation) and mechanical properties (duct-like secretory evacuation) are amenable to significantly furthering these efforts.

## 9. Applications of Materials for Osteoblast Differentiation and Bone Regeneration

The microenvironment of the mesenchymal stem cells strictly regulates their adhesion, proliferation, and differentiation. Previous studies have shown that small molecules and biomaterial scaffolds can induce the osteoblast differentiation and bone regeneration [215]. These studies have shown that markers for the osteoblast differentiation were increased in cells cultured on scaffold substrates. Several signaling pathways (BPMs, TGFβ, and Wnts) are involved in nanomaterial-induced stem cell differentiation towards osteoblast. Park et al. demonstrated that ε-aminocaproic acid/chitosan-incorporated nanoparticles in fibrin gel induce the osteoblast differentiation and bone regeneration [216]. Differentiation of stem cells can not only be induced by nanotopographical features, but also by the stiffness of the scaffold materials [217,218]. A recent study observed collagen-derived dipeptide prolyl-hydroxyproline (Pro-Hyp) promotes osteoblastic MC3T3-E1 cell differentiation and upregulation of osteogenic genes via FOXG1 expression [219]. Fu et al. demonstrated that poly(ε-caprolactone)-poly(ethylene glycol)-poly(ε-caprolactone) (PCL-PEG-PCL, PCEC) PCEC scaffold were optimal for cartilage tissue engineering as they provided optimal cell proliferation and adhesion for repair of cartilage defects [220]. In another study, chitosan/β-1,3-glucan/HA (chit/glu/HA) scaffold enhanced osteogenic differentiation via increasing TNF-α production, and could be a promising biomaterial for bone regeneration applications in specific clinical scenarios [221].

## 10. Future Perspectives

There have been many major developments in the field of biomaterials and nanotechnology that hold much promise for medical and dental clinical care in the near future. Thus far, many types of dental biomaterials have been engineered through nanotechnology, and several are already available for clinical use. Among them, superior cement and resin composites are being used for the reconstruction of missing tooth structures in dentistry. Bioglass nanoparticles have been incorporated into resin composite to mimic several natural characteristics of tooth structures, and to achieve long-term physical, mechanical, and biological properties, such as hardness, strength, toughness, and antimicrobial activity. Despite the rapid progress in production, properties, characterization, and application of small molecules and nanomaterials, there remain several challenges, such as safety regulations, ethics, and cost of these materials. Advanced drug delivery strategies for regenerative medicine represents a significant potential for a broad range of human diseases These materials essentially are attempting to mimic several complex physiological and pathophysiological processes. Learning from rigorous preclinical lab studies, single regenerative treatment strategies, such as delivery growth factors, cells, small molecules, peptides, and nucleotides have been noted to have significant limitations. An ideal therapeutic approach will likely be a multimodal approach, combining sophisticated fabrication and delivery systems with these singular approaches. These include the growing excitement with 3D bioprinting and several nanofibrous scaffold generation techniques. The growing evidence for smart systems that sense-and-respond to specific stimuli that are gaining from advances in biosensing, robotics, and artificial intelligence, are now able to improve monitoring and modulating biological response in a precisely controlled manner. The progress and accomplishments in biomaterials and biology are enabling the precision-medicine initiative to provide personalized, safe, and effective care.

## 11. Conclusions

Engineering precise cell–matrix interactions and providing extracellular biological cues can ensure regulated cellular responses, such as attachment and survival, expansion, and proliferation, migration, polarization, and patterning, as well as functional differentiation and tissue-organ functions. Various biomaterial formulations and fabrication techniques are enabling these efforts at hierarchical length scales for tissue regeneration. The use of controlled-release delivery systems, with both agonists and antagonists, have enabled development of discrete morphogen fields simulating embryonic development scenarios. These current advances and exciting ongoing progress are poised bring to fruition the promise of stem cell biology to clinical regenerative medicine.
